# School-based deworming programmes: Knowledge and perceptions regarding soil-transmitted helminth infections among schoolteachers in Tamil Nadu, India

**DOI:** 10.1371/journal.pgph.0004319

**Published:** 2025-03-31

**Authors:** Kumudha Aruldas, Rohan Michael Ramesh, Selvakumar Prasad, Gideon John Israel, Hugo Legge, Judd L. Walson, Arianna Rubin Means, Sitara Swarna Rao Ajjampur

**Affiliations:** 1 The Welcome Trust Research Laboratory, Division of Gastrointestinal Sciences, Christian Medical College, Vellore, Tamil Nadu, India; 2 Department of Disease Control, London School of Hygiene and Tropical Medicine, London, United Kingdom; 3 Department of International Health, Johns Hopkins University, Baltimore, Maryland, United States of America; 4 The DeWorm3 Project, University of Washington, Seattle, Washington, United States of America; 5 Department of Global Health, University of Washington, Seattle, Washington, United States of America; Kamuzu University of Health Sciences, MALAWI

## Abstract

In accordance with World Health Organization guidelines, the school-based deworming for soil-transmitted helminths (STH) is being implemented in India since 2015 through teachers on National Deworming Day (NDD). This study aimed to assess teachers’ knowledge levels and perception of STH and NDD programmes in southern India using a cross-sectional study design with purposive sampling of teachers involved in the NDD programme. Data from 402 teachers across 221 schools were analysed using STATA 16.0 software. Overall, 37% of the teachers from 52% of schools had attended an NDD training programme. Of a maximum possible score of 32, the knowledge levels were categorised as low (<50%), average (at least 50%), and adequate (70% and above). While 84% of the teachers had an average level of knowledge, only 33% had an adequate level of knowledge. The results showed significant knowledge gaps regarding important aspects of the STH programme including, helminths species being treated, age eligibility of children for treatment, and the name of and side-effects of the deworming tablet distributed. Adequate level of knowledge was more likely among teachers from government schools than those from government-aided/private schools (OR: 3.74; CI: 1.80 – 7.74; p<0.001); those who attended an NDD training programme than those who never attended (OR: 2.69; CI: 1.73 – 4.19; p<0.005); and female teachers compared to male teachers (OR: 1.94; CI: 1.22 - 3.08; p<0.005). A weak but significant positive correlation was observed between knowledge levels and perception (Spearman’s rho=0.2075; p=0.002). Teachers from all schools must be encouraged to participate in formal STH training programmes coordinated by the government, and these programmes should be evaluated periodically for effectiveness.

## Introduction

The World Health Organization (WHO) estimated the burden of soil-transmitted helminths (STH) as 820 million people infected with *Ascaris lumbricoides* (roundworm), 460 million with *Trichuris trichiura* (whipworm), and 440 million with *Ancylostoma duodenale* and *Necator americanus* (hookworms) [1]. The impact of STH control programmes from 2000 – 2019 showed a 53% reduction in disability-adjusted life year lost due to STH [2]. In South Asia, an estimated 361 million people were infected with at least one STH species [3]. The WHO Roadmap 2030 for Neglected Tropical Diseases (NTD) set the goal of eliminating STH-related morbidity by ensuring 75% coverage of targeted populations with deworming, including pre-school-aged children (PSAC: 1–4 years) and school-aged children (SAC: 5–14 years), and women of reproductive age [4]. In India, an estimated 258 million individuals are infected by STH contributing to 21% of the global STH burden [3].

Teachers have played a crucial role in school-based deworming programmes in many countries. In a study from the Philippines, 42% of the teachers had stated that STH was a major problem as open defaecation was common in their communities, and they showed a favourable attitude towards mass drug administration (MDA) [5]. Teachers in a Bangladesh study had reported that walking barefoot or playing with soil (78%) and lack of handwashing with soap (52%) as reasons for STH transmission and that transmission could be prevented by deworming (97%) and health education (88%) [6]. A Malaysian study reported a low knowledge level among teachers regarding types of STH (roundworm: 32%, hookworm: 36%, and whipworm: 0%); that STH spread by walking barefoot (28%) and from dirty hands (52%); and STH could be prevented by deworming (44%), hand washing (32%), and wearing shoes (28%) [7].

The Ministry of Health and Family Welfare (MoHFW), Government of India, has been implementing biannual school-based deworming programme, the National Deworming Day (NDD) programme, for PSAC and SAC through schools and *anganwadi* centres (government-run community-based pre-schools for children between 3-6 years of age) [8]. This NDD programme was initiated in 11 states in 2015 and was expanded to include all 36 states of India in 2016. The MoHFW organises training programmes for teachers before every round of the NDD programme, which includes information on the benefits of deworming, administration of Albendazole tablets, management of adverse events, reporting coverage, and logistics and supply chain management [8]. The NDD tool kit which includes Information, Education, and Communication (IEC) materials such as posters, banners, handouts, frequently asked questions, adverse event management guidance notes, and reporting formats is distributed to the teachers and disseminated in all schools. Teachers are expected to conduct IEC activities within the school premises and to interact with parents regarding the NDD programme [8]. Teachers are the foundation of school-based NDD programme in India and are required to ensure high coverage of school-based deworming. However, there is limited literature from India on teachers’ knowledge and perceptions of STH and the NDD programme. As they are expected to interact with parents and are key community influencers in general, it is important to understand their knowledge level and perception of STH and deworming to optimise training programmes and potentially increase coverage.

The DeWorm3 Trial, a community-based cluster-randomised trial, also implemented in Benin and Malawi, was carried out to assess the feasibility of interrupting STH transmission by comparing biannual community-wide MDA (cMDA) with the standard of care, school-based deworming programme [9,10]. This study among the teachers aimed to assess the knowledge and perception of STH infections and control programmes among teachers and differences, if any, in STH knowledge levels between teachers in the intervention and control clusters of the DeWorm3 project field sites in India.

## Materials and methods

### Ethics statement

Ethical approval for this research was received from the Human Subjects Division at the University of Washington (STUDY00000180) and the Institutional Review Board at Christian Medical College, Vellore as an amendment to the DeWorm3 study in India (10392 [INTERVEN]; IRB – A09, August 26, 2020). Informed written consent was obtained from all the teachers who participated in the survey.

### Study design and setting

**Fig 1:**
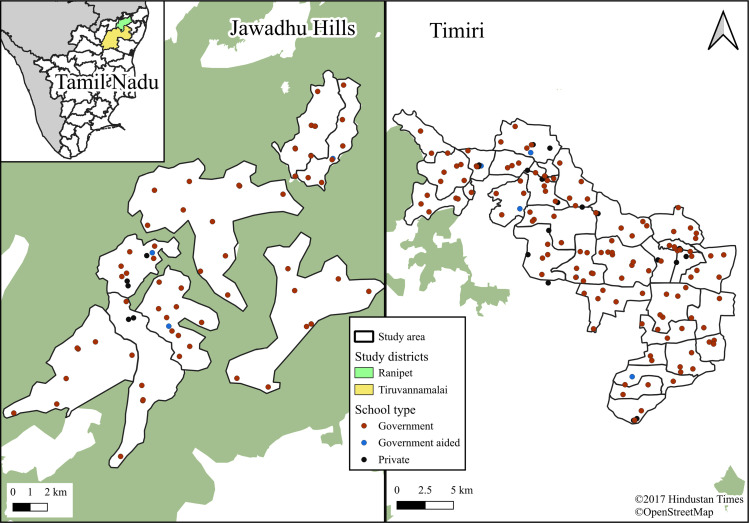
Study area with types of schools. [Sources and copyright information: 1) State and district shapefiles: HindustanTimesLabs, available at GitHub repository (1) - https://github.com/HindustanTimesLabs/shapefiles/tree/master/india. Licensed underhttps://github.com/HindustanTimesLabs/shapefiles/blob/master/LICENSE and 2) Forest layer: OpenStreetMap contributors, available at https://www.openstreetmap.org/. Used under the Open Database License (ODbL)https://www.openstreetmap.org/copyright].

Using a cross-sectional study design, this survey was conducted in the state of Tamil Nadu in southern India in two blocks, the Timiri block in Ranipet district and the Jawadhu Hills block in Tiruvannamalai district, in the state of Tamil Nadu in southern India where the DeWorm3 project was being implemented [11] “Fig 1”.

### Sample size

The sample size was calculated using the formula,

n= N/(1+Ne^2^), where N is the population size (1,420), e is the margin of error (5%). Including a design effect of 1.2 and 10% non-response rate, the estimated sample size (n) was 412.

### Study population and sampling

The study population included 1,420 teachers from 221 schools (109 in intervention and 112 in control clusters). Two teachers per school, as identified by the school principal (school administrator) as being involved in the NDD programme at their school, were purposively selected to participate in the survey. From the 40 primary schools that had only one teacher, all were invited to participate.

### Data collection

In the absence of an existing comprehensive questionnaire that included questions related to STH knowledge, perception, and treatment, we developed a semi-structured questionnaire with 35 questions (S1 Text). This questionnaire included 10 questions about the background characteristics of the teachers, nine open-ended questions to assess knowledge of STH and NDD programmes, and 16 Likert scale questions on perceptions about STH infections scored on a 5-point scale ranging from ‘strongly agree’ to ‘strongly disagree’. Examples of STH perceptions include STH infections among adults, treatment of those not at risk of STH infections, and safety and effectiveness of deworming tablets. The questionnaire was translated into the local language (Tamil) and pilot tested before data collection. A unique identification number was allotted to each participating teacher, and no personal identification details were collected. Between 24/08/2022 and 29/09/2022, trained field workers approached the school administrator and requested them to identify two teachers involved in the school-based deworming programme at their school for participation in the survey. Then, they met the identified teachers within the school premises, obtained their informed written consent, and requested them to complete the paper-based self-administered questionnaire. The field workers waited until the teachers completed the questionnaire and collected them.

### Data management and analysis

The data were double entered in the data entry forms prepared on SurveyCTO (Dobility, Inc., Cambridge, MA, and Ahmedabad, India), and mismatched entries were verified with the hard copy and finalised. For the knowledge questions, we assigned a score of 2 for each correct response and 0 for each incorrect response; thus, the maximum possible knowledge score was 32. In the absence of literature on knowledge scores among teachers, we considered a score of less than 16 (<50%) as ‘low’, 16 and above (at least 50%) as ‘average’, and 22 and above (at least 70%) as ‘adequate’ knowledge. The responses to 5-point Likert scale questions were scored from 5 to 1, with 5 being the most positive response and 1 being the most negative response; thus, the maximum possible positive perception score was 65. The internal consistency of the questionnaire used for data collection showed a Cronbach’s alpha reliability coefficient of 0.66.

The data was analysed using STATA 16.0 software (StataCorp, TX, USA). Descriptive analysis of frequency and percentage distribution with Chi‑square test and Fisher’s exact test were used to examine the differences between intervention and control clusters. Mean scores and standard deviation (SD) and univariable and multivariable logistic regression analyses were used to assess factors associated with average and adequate knowledge levels. Spearman’s rank correlation coefficient (Spearman’s rho) was used to understand the relationship between knowledge levels and positive perception about STH and treatment. Statistical significance was defined at a p-value of <0.05.

## Results

### Background characteristics of the participants

A total of 402 teachers from 221 schools participated in the survey. Most of the teachers were women (61%) and the mean age was 44.3 years (SD: 9.65). They were educated to the postgraduate level (75%), had taught science and/or environmental science (84%), and had 10 or more years of teaching experience (71%) with 17.1 mean years of teaching (SD: 8.93) (S1 Table). Most of them were aware of the school-based NDD programme (98%) and DeWorm3 project related to cMDA in their area (80%). Participants who had ever attended a NDD training (37%) were from 52% of the study schools. Other than gender, there was no significant difference in the background characteristics of teachers from schools situated in the intervention and control clusters (S1 Table).

### Signs and symptoms, spread, and prevention about STH infections

**Fig 2:**
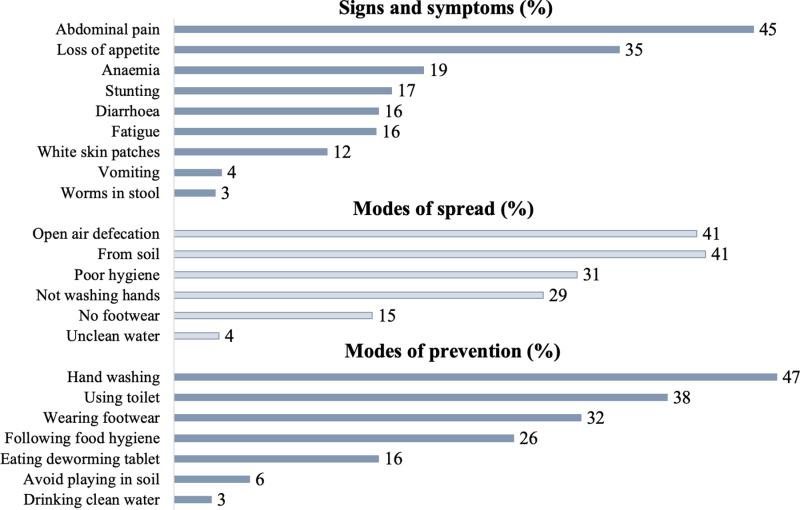
Proportion of teachers correctly identifying STH signs & symptoms and modes of spread and prevention (n = 402).

The two main signs and symptoms the teachers reported were ‘abdominal pain’ and ‘loss of appetite’; two main modes of spread reported were ‘from soil’ and ‘due to open defecation’; and two main modes of prevention reported were ‘hand washing’ and ‘use of toilets’ “Fig 2”.

### Knowledge of STH and NDD programme

**Fig 3:**
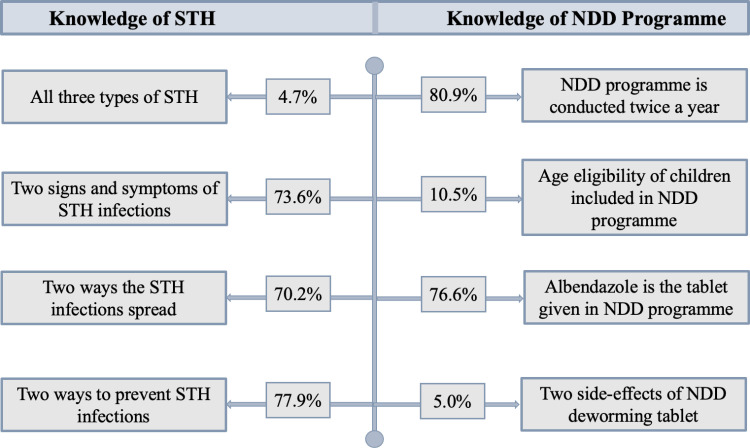
Proportion of teachers responding correctly to specific indicators of STH and NDD programmes (n = 402).

Most of the teachers agreed or somewhat agreed that they knew enough about STH (97%), however, only a few of them (5%) could name all three types of STH (hookworm, roundworm, and whipworm) “Fig 3”. Most teachers listed hookworm (85%) but fewer listed roundworm and whipworm (34% and 11%, respectively) and many incorrectly listed tapeworms as one of the STH infections (73%). Many of them (77%) knew that albendazole was the deworming tablet distributed in the NDD programme and is distributed twice a year (81%). Very few correctly stated the lower and upper age of children eligible to receive a deworming tablet in the NDD programme (11%) and described at least two side-effects of albendazole (5%).

### Perception about STH infections

Many teachers believed that adults living in the villages around their schools could be STH infected (62%) and that those who always use a toilet (86%) and wash their hands with soap before eating (92%) will not have intestinal worms. Some of them believed that STH infections would be more common among children under five years of age as compared to older children (17%) and among women compared to men (26%). Several of them perceived STH infections as minor infections that caused no harm to the body (45%) and that the STH infections could get cleared even without treatment (44%) or that intestinal worms help in digestion (17%).

Nearly all teachers perceived that STH infections could be controlled by treating the children (95%). Many of them believed that all adults need not be treated (40%) and that only those who feel they could be STH-infected need to be treated (38%). They reported that the deworming tablet had side-effects (65%) and had expressed concerns about the side-effects of the deworming tablet distributed to children (47%). Most teachers perceived that the tablets distributed in the NDD programmes were effective (98%) and safe (96%) but also believed that traditional remedies were very effective in treating worms (82%).

### STH knowledge scores and associated factors

**Fig 4:**
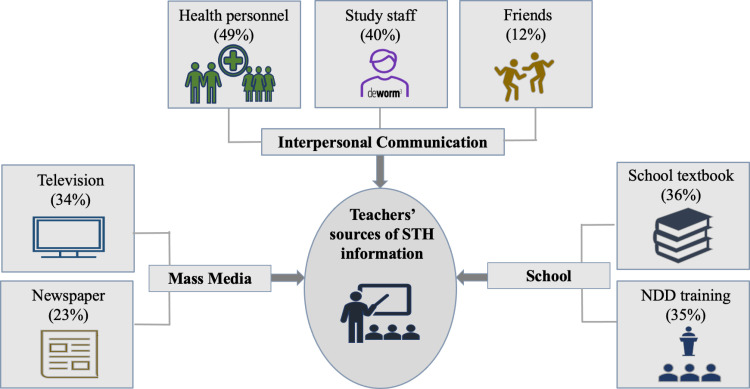
Teachers’ sources of information about STH infections (n = 402).

The common sources of STH knowledge reported by the teachers were from health personnel (49%), DeWorm3 project staff (40%), school textbooks (36%), NDD training (35%), television (34%), newspaper (23%), and friends (12%) “Fig 4”.

The mean knowledge score was 18.81 (SD: 4.39; range: 2-26). There was no significant difference in mean knowledge scores between male and female teachers (males – mean: 18.20, SD: 4.48; range: 2-26; and females – mean: 19.19, SD: 4.29, range: 2-26; P = 0.713). Similarly, there was no significant difference in mean knowledge scores between teachers from intervention and control clusters (intervention clusters – mean: 18.98, SD: 4.28, range: 2-26; and control clusters – mean: 18.63, SD: 4.49, range: 2-26; P = 0.638). Few of them had a ‘low’ knowledge level (16%) and while most of them had an ‘average’ knowledge level (84%) only 33% had an ‘adequate’ knowledge level (score of 70% or more). The univariable logistic regression analysis showed that adequate knowledge was associated with age, type of school, years of teaching, teaching science and/or environmental science, and having attended NDD training (Table 1). The multivariable analysis showed that ‘adequate’ knowledge was nearly four times more likely among those teaching in government schools compared to those teaching government-aided/private schools (OR: 3.74; CI: 1.80 – 7.74; p<0.001); three times more likely among those having attended NDD training compared to those who never attended NDD training (OR: 2.69; CI: 1.73 – 4.19; p<0.005); and two times more likely among female teachers compared to male teachers (OR: 1.94; CI: 1.22 - 3.08; p<0.005) (Table 1).

**Table 1:** Factors associated with STH knowledge.

Characteristics	Average Knowledge						Adequate Knowledge					
	Knowledge Score		Univariable		Multivariable		Knowledge Score		Univariable		Multivariable	
	<16 (n, %)	>=16 (n, %)	OR (CI)	P- value	OR (CI)	P- value	<22 (n, %)	>=22 (n, %)	OR (CI)	P- value	OR (CI)	P- value
**Age**												
20–30	16 (24.2)	16 (4.8)	Ref		Ref		28 (10.5)	4 (3.0)	Ref			
31–40	18 (27.3)	105 (31.2)	5.83 (2.48–13.71)	**<0.001**	5.22 (2.07–13.16)	**<0.001**	85 (31.7)	38 (28.4)	3.13 (1.03–9.55)	**0.045**	**–**	**–**
41–50	13 (19.7)	82 (24.4)	6.31 (2.55–15.62)	**<0.001**	4.52 (1.69–12.09)	**0.003**	60 (22.4)	35 (26.1)	4.08 (1.32–12.61)	**0.014**	**–**	**–**
>50	19 (28.8)	133 (39.6)	7 (3.01–16.27)	**<0.001**	4.35 (1.63–11.58)	**0.003**	95 (35.5)	57 (42.5)	4.2 (1.4–12.59)	**0.010**	**–**	**–**
**Gender**												
Male	29 (43.9)	128 (38.1)	Ref		Ref		114 (42.5)	43 (32.1)	Ref		Ref	
Female	37 (56.1)	208 (61.9)	1.27 (0.75–2.17)	0.374	1.74 (0.96–3.16)	0.069	154 (57.5)	91 (67.9)	1.57 (1.01–2.42)	**0.044**	1.94 (1.22–3.08)	**0.005**
**Teacher’s level of education**												
Higher secondary	8 (12.1)	24 (7.1)	Ref				22 (8.2)	10 (7.5)	Ref			
Graduate	12 (18.2)	58 (17.3)	1.61 (0.58–4.44)	0.356	–	–	48 (17.9)	22 (16.4)	1.01 (0.41–2.48)	0.986	–	–
Postgraduate	46 (69.7)	254 (75.6)	1.84 (0.78–4.35)	0.164	–	–	198 (73.9)	102 (76.1)	1.13 (0.52–2.48)	0.755	–	–
**Level of schools**												
Primary school	44 (66.7)	221 (65.8)	Ref				169 (63.1)	96 (71.6)	Ref			
Middle school	9 (13.6)	68 (20.2)	1.50 (0.69–3.24)	0.297	–	–	53 (19.8)	24 (17.9)	0.79 (0.46–1.37)	0.414	–	–
High school and above	13 (19.7)	47 (14.0)	0.71 (0.36–1.44)	0.353	–	–	46 (17.2)	14 (10.5)	0.54 (0.28–1.02)	0.059	–	–
**Type of school teaching in**												
Government aided/private	20 (30.3)	50 (14.9)	Ref		Ref		60 (22.4)	10 (7.5)	Ref		Ref	
Government	46 (69.7)	286 (85.1)	2.48 (1.36–4.55)	**0.003**	1.74 (0.83–3.62)	0.141	208 (77.6)	124 (92.5)	3.58 (1.77–7.24)	**<0.001**	3.74 (1.80–7.74)	**<0.001**
**Years of teaching experience**												
<=10	28 (42.4)	94 (27.9)	Ref				88 (32.8)	34 (25.4)	Ref			
11–20	23 (34.9)	99 (29.5)	1.28 (0.69–2.38)	0.432	–	–	90 (33.6)	32 (23.8)	0.92 (0.52–1.62)	0.773	–	–
21–30	12 (18.2)	114 (33.9)	2.83 (1.36–5.87)	**0.005**	–	–	68 (25.4)	58 (43.3)	2.21 (1.30–3.75)	**0.003**	–	–
>=31	(4–6)	29 (8.6)	2.88 (0.82–10.16)	0.100	–	–	22 (8.2)	10 (7.5)	1.18 (0.50–2.74)	0.706	–	–
**Teaching either science or environmental science or both**												
No	16 (24.2)	47 (14.0)	Ref				50 (18.7)	13 (9.7)	Ref			
Yes	50 (75.8)	289 (86.0)	1.97 (1.04–3.74)	**0.039**	–	–	218 (81.3)	121 (90.3)	2.13 (1.12–4.09)	**0.022**	–	–
**Aware of National Deworming Day programme**												
No	3 (4.5)	7 (2.1)	Ref				9 (3.4)	1 (0.8)	Ref			
Yes	63 (95.5)	329 (97.9)	2.24 (0.56–8.89)	0.252	–	–	259 (96.6)	133 (99.3)	4.62 (0.58–36.87)	0.149	–	–
**Ever attended National Deworming Day training**												
No	50 (75.8)	202 (60.1)	Ref		Ref		190 (70.9)	62 (46.3)	Ref		Ref	
Yes	16 (24.2)	134 (39.9)	2.07 (1.13–3.79)	**0.018**	1.70 (0.88–3.29)	0.117	78 (29.1)	72 (53.7)	2.83 (1.84–4.35)	**<0.001**	2.69 (1.73–4.19)	**<0.001**
**Heard about community-wide deworming programme (DeWorm3)**												
No	15 (22.7)	65 (19.4)	Ref				59 (22.1)	21 (15.7)	Ref			
Yes	51 (77.3)	271 (80.7)	1.23 (0.65–2.32)	0.53	–	–	209 (77.9)	113 (84.3)	1.52 (0.88–2.63)	0.135	–	–
**Type of DeWorm3 trial arm**												
Control cluster	36 (54.5)	167 (49.7)	Ref		Ref		142 (53.0)	61 (45.5)	Ref			
Intervention cluster	30 (45.5)	169 (49.5)	1.21 (0.72–2.06)	0.472	1.14 (0.65 -1.99)	0.639	126 (47.0)	73 (54.5)	1.35 0.89–2.04)	0.159	–	–

OR - Odds ratio; CI – 95% Confidence interval.

**Fig 5:**
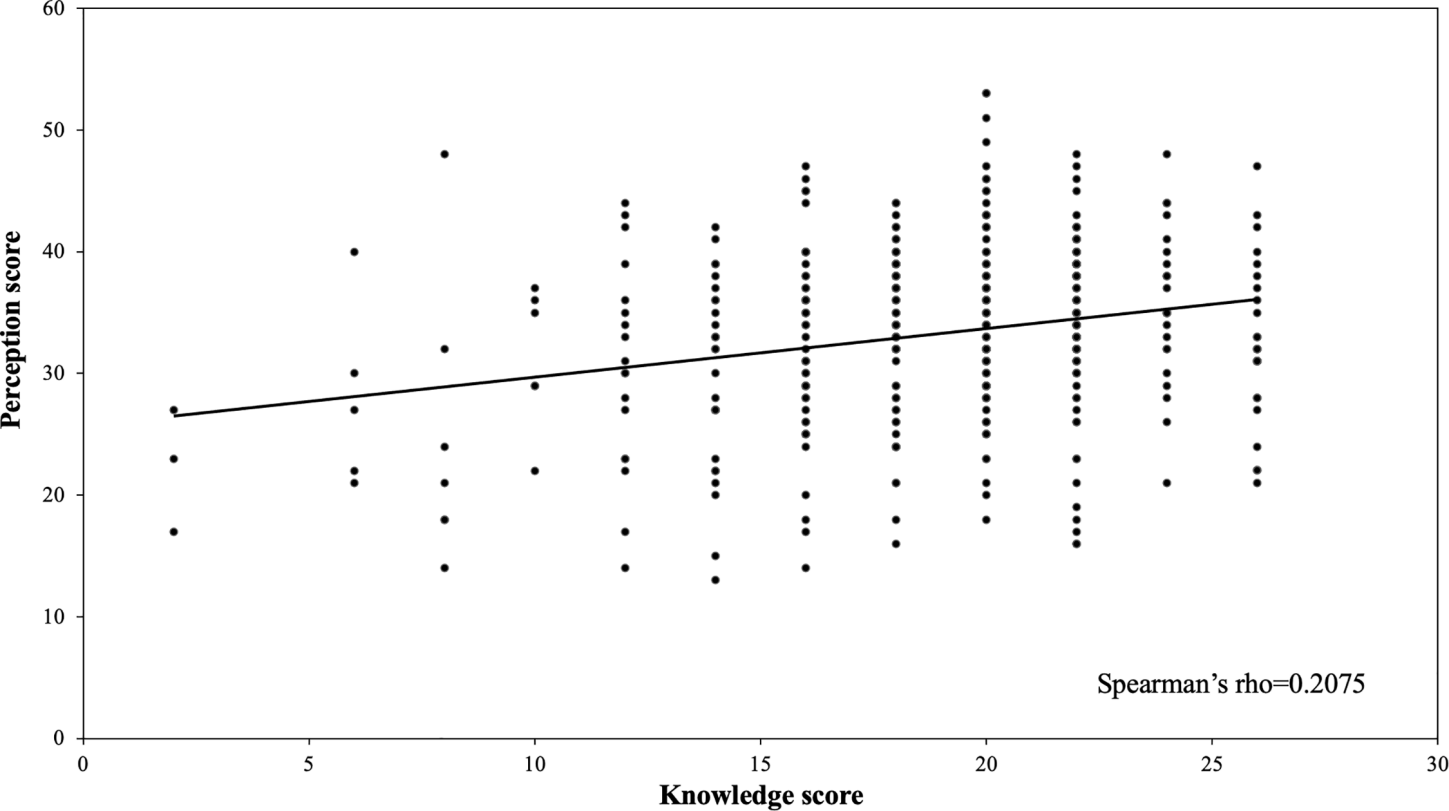
Correlation between knowledge score and perception score.

The mean score reflecting positive perceptions towards controlling STH infections was 34 (SD: 7.34; range: 13-53). The Spearman’s rank correlation coefficient test showed a significant positive but weak correlation between knowledge and perceptions towards STH infections and treatment (Spearman’s rho=0.2075; p=0.002) “Fig 5”. The results showed that as the knowledge score increased, the positive perception score also increased.

## Discussion

This study aimed to understand knowledge and perceptions about STH and the NDD programme among teachers involved in school-based deworming in southern India. The survey results demonstrate that only one-third of the teachers qualified as having an ‘adequate’ level of knowledge, defined as a score of 70% or more. Knowledge was specifically low regarding STH species treated, the age eligibility of children for treatment, and the name and side-effects of the tablet distributed in the NDD programme. They believed that STH infections could be controlled by treating only children, and only symptomatic adults need to be treated. Factors associated with ‘adequate’ knowledge among teachers were teaching at government schools, attended a NDD training, and female teachers. Many teachers had mistakenly considered tapeworms as an STH like the community drug distributors (22%) residing in the same geographical area had considered earthworms as an STH [12].

The teachers harboured the misconception that intestinal worms would aid in digestion. Similar beliefs were reported in a qualitative study of the community members who were living in the same geographical area as the teachers [13]. A study in Bangladesh showed that 20% of the community members believed that intestinal worms and therefore, it would be bad for health to eliminate all the worms [14]. Community members in China also believed that intestinal worms are essential for digestion, and they become a health issue only when the worms grew “very big” [15]. The teachers of our study had expressed fear of the side-effects of albendazole that they distributed at schools. Similarly, in a study from the Philippines, over 80% of the teachers had reported being worried about managing the side-effects of deworming tablets and 90% of the parents feared that the teachers would not detect and manage the side-effects of deworming tablets and would not prefer teachers administering the deworming tablets (37%) [5]. Therefore, such myths and misconceptions and gaps in knowledge should be addressed in the teachers’ training programmes to attain high coverage of school-based deworming programmes.

The teachers of our study had perceived that those who always use toilets and wash their hands with soap before eating may not be STH infected and STH infections could get cleared with traditional remedies and sometimes even without any treatment. A qualitative study among the community members in Benin, India, and Malawi showed that those who considered themselves at low risk of STH may not participate in MDAs and were concerned about treating people who may not be infected [16]. Such perceptions affect MDA programmes as a study on lymphatic filariasis in India showed a gap between MDA distribution of 74% and a compliance rate of 59% [17]. Our study showed that the likelihood of adequate knowledge level was higher among those who attended an NDD training programme; therefore, the ‘training for teachers’ organized by governments prior to executing school-based deworming programmes could be an optimal platform to boost knowledge levels and discuss positive perceptions about STH programmes. Pre-training assessment tools would help identify and address knowledge and perception gaps when participation may vary in each training round. This study showed low participation of schools in NDD training programmes, specifically the government-aided and private schools, which were also less likely to have teachers with an adequate knowledge level of STH and NDD programme. In 29 out of the 38 schools included in a Kenyan study, fellow teachers often facilitated and supported the teachers involved in drug distribution and record keeping [18]. Therefore, ensuring the participation of all schools and training all teachers, irrespective of their role in school-based deworming programmes, would be beneficial.

Similar to a study from the Philippines where health workers were the main source of STH information for nearly two-thirds of the teachers, this study also showed that inter-personal communication with health workers was the most common source of information for the teachers [5]. This study also showed that female teachers were more likely to have adequate levels of knowledge than male teachers. A similar observation was made among community members living in the same geographical area where women had higher STH knowledge than men, possibly because of their interactions with health workers of maternal and child health programmes [14]. A study in Malaysia showed that compared to a control group, teachers who were trained using a health education learning package that included a guidebook to STH infections, posters, a comic book, a music video, a puppet show, drawing activities and an aid kit had a higher level of STH knowledge [7]. Improving perceptions and knowledge requires sustained and intensive educational efforts and is crucial as knowledge does not necessarily lead to behaviour change [19]. The policymakers from other states of India have reported that engaging with local leaders and community groups is crucial for the successful implementation of the MDA programme [20]. In a study from the Philippines, the local officials had reported monitoring of school-based deworming as crucial for achieving high MDA coverage [21].

The significant strength of this study is that it included teachers from all types of schools - government schools, government-aided schools, and private schools. However, the generalizability of the results may be limited because we used a purposive sampling method where school administrators identified the teachers involved in the Indian NDD programme to participate in this study. It is also possible that the school administrators may have introduced selection bias and identified teachers they knew were more knowledgeable about STH and NDD programmes to participate in this study.

## Conclusion

This study showed lower levels of adequate STH knowledge among schoolteachers involved in the school-based deworming programme in southern India. This study identified key knowledge gaps related to treatment eligibility criteria and side-effects and identified perception gaps that would be important to address and optimize during STH training programmes. These results highlight the importance of including teachers from all types of schools in STH training programmes, designing and reviewing training modules to ensure that they adequately address misconceptions, and the need for continuous evaluation of STH training programmes.

## Supporting information

S1 Text Study questionnaire. (PDF)

S1 Table Background characteristics of teachers participating in survey. (DOCX)
