# Supplementary material for: School-based deworming programmes: Knowledge and perceptions regarding soil-transmitted helminth infections among schoolteachers in Tamil Nadu, India
Source: PLOS Glob Public Health. 2025 Mar 31;5(3):e0004319. doi: 10.1371/journal.pgph.0004319 (PMC11957371; doi:10.1371/journal.pgph.0004319)
Supplement: S1 Text — (PDF) [file pgph.0004319.s001.pdf]

Study Questionnaire School ID: _________ Date: ______________

Please circle or indicate your response

S. No.	Statement	Response
1.	I have heard of NDD (National Deworming Day) program for intestinal worms.	Yes / No
2.	I have attended NDD training program.	Yes / No
2.a	(If Yes):<br>Totally, so far, I attended ___________ NDD training programs.	
3.	**Please circle only one option for the statement**<br>I have enough information about intestinal worms that is needed to tell the school children. - (**please circle only one option**)<br>1. I Agree 2. Somewhat agree 3. Not sure 4. Somewhat disagree 5. I disagree	
4.	I learnt about soil-transmitted intestinal worms **mainly** from – (**can circle more than one option)**<br>1. NDD training 2. Internet 3. Newspaper 4. Television 5. School textbook 6. CMC staff 7. Health personnel 8. Friends<br>9. Any other way (specify)__________________	
5.	In the NDD program, deworming tablet is given ________ times in a year	
6.	Children from the ages of _____________ years to ________________ years can take the deworming tablet given in NDD program	
7.	In my opinion, out of 100 children in my school, about _______children may have intestinal worms.	
8.	I have heard of deworming program where all adults are also given deworming tablet.	Yes / No

For the following statements, **please tick (✓) only one option:**

No	Statements	Options					
		**(a) Agree**	**(b) Somewhat agree**	**(c) Neither agree nor disagree**	**(d) Somewhat disagree**	**(e) Disagree**	**(f) Do not know**
9.	Those who always wash their hands with soap before eating will not have intestinal worms						
10.	Those who always use toilets will not have intestinal worms						
11.	Even without treatment, there is a chance for worms can get cleared from the intestines						
12.	Only those who feel they have intestinal worms need to eat deworming tablet, not others						
13.	Only small children under 5 years of age have Intestinal worms, not the older children						
14.	Deworming tablet distributed to children is an effective drug						
15.	Traditional medicines are also very effective in treating intestinal worms						
16.	Intestinal worms help in digesting the food						
17.	Intestinal worms are minor infections that cause no harm to the body						
18.	Deworming tablet distributed to children is a safe drug						
19.	In my opinion, spread of intestinal worms can be effectively controlled by continuously deworming the children						
20.	It is not necessary to give deworm tablets to all adults in the village to control spread of intestinal worms in children						
21.	Intestinal worms are common in adults who live in the villages nearby this school						
22.	Intestinal worms are more among women than men						
23.	There are side-effects of the deworming tablets distributed in schools						
24.	I am worried about side-effects of the deworming tablet when distributed to children						

25.	The soil-transmitted intestinal worms are - (**can circle more than one option**)<br><br>1. Tapeworm 2. Whipworm 3. Hookworm 4. Roundworm 5. Pinworm 6. Flatwork 7. Thread worm<br><br>8. Any other worm (specify)__________________
26.	Does the tablet distributed in NDD program have side-effects?<br>Yes / No / Do not know
26a	If yes, two main side-effects of deworming tablet distributed in NDD program are<br><br>1. ____<br><br>2. ____
27.	Two **main** ways by which soil-transmitted intestinal worms can spread are -<br><br>1. ____<br><br>2. ____
28.	Two **major** signs and symptoms of intestinal worms are -<br><br>1. ____<br><br>2. ____
29.	Two **main** ways of preventing soil-transmitted intestinal worms are -.<br><br>1. ____<br><br>2. ____
30.	Name of the tablet given to children in NDD program is ____________________

**Background characteristics**		
31.	My age is ___________ years	32. I am — Male / Female
33.	My highest education is – HSC (D.TEd) / BA / BSc / BCom / MA / MSc / MCom other (specify) ________________<br><br>33.a My major subject in UG was ___________________________<br><br>33.b My major subject in PG was ________________________	
34.	Total number of years I have been working as a teacher. _______________ Years	
35.	I have taught these subjects:<br>1. Science 2. Environmental science 3. Neither science nor environmental science<br><br>35.a. I have taught the above-mentioned subjects for ____________ years	

Thank you very much for your time and opinion.

Cluster name: ___________________________________ Cluster number: ___________

Type of school: Government school / Government aided school / Private school

Level of school: Primary School (1-5) / Middle School (1-8) / High School (6-10) / Higher Secondary School (6-12)
