# Supplementary material for: School-based deworming programmes: Knowledge and perceptions regarding soil-transmitted helminth infections among schoolteachers in Tamil Nadu, India
Source: PLOS Glob Public Health. 2025 Mar 31;5(3):e0004319. doi: 10.1371/journal.pgph.0004319 (PMC11957371; doi:10.1371/journal.pgph.0004319)
Supplement: S1 Table — (DOCX) [file pgph.0004319.s002.docx]

**S1 Table: Background characteristics of teachers participating in survey**

| **Characteristics** | **Overall** | | **DeWorm3 Intervention cluster** | | **DeWorm3 Control cluster** | | **Chi-square test** |
| --- | --- | --- | --- | --- | --- | --- | --- |
|  | **n = 402** | **%** | **n = 199** | **%** | **n=203** | **%** | **P-value** |
| **Age** | | | | | | | |
| 20 - 30 | 32 | 8.0 | 14 | 7.04 | 18 | 8.9 | 0.899 |
| 31 - 40 | 123 | 30.6 | 61 | 30.7 | 62 | 30.5 |  |
| 41 - 50 | 95 | 23.6 | 49 | 24.6 | 46 | 22.7 |  |
| > 50 | 152 | 37.8 | 75 | 37.7 | 77 | 37.9 |  |
| **Gender** | | | | | | | |
| Male | 157 | 39.1 | 67 | 33.7 | 90 | 44.3 | **0.028** |
| Female | 245 | 61.0 | 132 | 66.3 | 113 | 55.7 |  |
| **Teacher’s level of education** | | | | | | | |
| Diploma-Teacher Education | 32 | 8.0 | 13 | 6.5 | 19 | 9.4 | 0.550 |
| Graduate | 70 | 17.4 | 34 | 17.1 | 36 | 17.7 |  |
| Postgraduate | 300 | 74.6 | 152 | 76.4 | 148 | 72.9 |  |
| **Years of teaching in schools** | | | | | | | |
| <=10 | 122 | 30.4 | 56 | 28.1 | 66 | 32.5 | 0.624 |
| 11 - 20 | 122 | 30.4 | 66 | 33.2 | 56 | 27.6 |  |
| 21 - 30 | 126 | 31.3 | 62 | 31.2 | 64 | 31.5 |  |
| > 30 | 32 | 8.0 | 15 | 7.5 | 17 | 8.4 |  |
| **Teaching either science or environmental science or both** | | | | | | | |
| Yes | 339 | 84.3 | 169 | 84.9 | 170 | 83.7 | 0.745 |
| No | 63 | 15.7 | 30 | 15.1 | 33 | 16.3 |  |
| **Aware of National Deworming Day program** | | | | | | | |
| Yes | 392 | 97.5 | 195 | 98.0 | 197 | 97.0 | 0.751 |
| No | 10 | 2.5 | 4 | 2.0 | 6 | 3.0 |  |
| **Ever attended National Deworming Day training** | | | | | | | |
| Yes | 150 | 37.3 | 74 | 37.2 | 76 | 37.4 | 0.958 |
| No | 252 | 62.7 | 125 | 62.8 | 127 | 62.6 |  |
| **Heard about community-wide deworming program (DeWorm3)** | | | | | | | |
| Yes | 322 | 80.1 | 166 | 83.4 | 156 | 76.9 | 0.099 |
| No | 80 | 19.9 | 33 | 16.6 | 47 | 23.1 |  |
| *Fisher’s exact | | | | | | | |
